# Design of Urban Public Spaces: Intent vs. Reality

**DOI:** 10.3390/ijerph15040816

**Published:** 2018-04-21

**Authors:** Mikkel Hjort, W. Mike Martin, Tom Stewart, Jens Troelsen

**Affiliations:** 1 Department of Sport Science and Clinical Biomechanics, Southern University of Denmark, Campusvej 55, Odense M, 5230, Denmark; jtroelsen@health.sdu.dk; 2 Architecture Department, University of California-Berkeley, 230 Wurster Hall #1820, Berkeley, CA 94720–1820, USA; wmmartin@berkeley.edu; 3 Human Potential Centre, Auckland University of Technology, AUT Millennium, Room SA225, New Zealand; tom.stewart@aut.ac.nz

**Keywords:** activating architecture, physical activity, transparent design, evidence based design, interdisciplinary collaboration, SOPARC

## Abstract

This study investigated how two public spaces for sport and recreation were utilized by different user groups, and how this aligned with the initial design objectives for these spaces. Two newly built urban spaces situated in Copenhagen, Denmark, provided the context for this investigation. The System for Observing Play and Recreation in Communities (SOPARC) was used to examine the physical activity of users in these two urban spaces. The architects responsible for designing each space were interviewed to ascertain the intended target group of each space and to unravel the reasons behind the design decisions. The SOPARC observations revealed that males were more vigorously active than females when using the recreation facilities, and the observed users did not align with the intended target groups. The interviews suggested that design decisions were based on minimal interdisciplinary knowledge, and that expert knowledge was chosen randomly. These findings point to a systematic lack of evidence-based practice when designing sport and recreational facilities. This article has implications for landscape architects and urban planners; a new method must be developed to embed interdisciplinary knowledge in the planning process of future sport and recreation projects. This must be done in a systematic way to make the design process transparent.

## 1. Introduction

Activating architecture has become part of the current dialog within environmental design professions [1]. The concept of activating architecture is grounded in the notion that physical environments, in this case urban sport environments, should engage the user both physically and psychologically. This concept is connected to an emergent health discourse: new buildings and landscapes have significant health-related responsibilities. The physical attributes of an environment can stimulate the movement of users, increase self-awareness and capacity, and contribute to higher public health goals. Residential urban environments have a significant impact on how physically active people are [2]. Research has shown that physical inactivity is one of the leading risk factors for lifestyle-diseases. It is estimated that physical inactivity causes 6–10% of premature mortality worldwide [3]. Lifestyle diseases such as obesity, type 2 diabetes, and cardiovascular disease continue to rise in Western countries [4,5]. This has increased the demand for designers and developers to illustrate how new urban spaces and facilities for physical activity and sport contribute to public health outcomes.

Urban design, transportation systems, and urban spaces are known predictors of physical activity [6], which can be conceptualised through the ecological model of active living [7]. This theoretical framework illustrates interdependent factors that influence physical activity behaviour across the four domains of active living: recreation, transportation, household, and occupation. Physical activity in each of these domains relies on different factors, such as individual skills, the social/cultural environment, the built environment, and the policy environment. It is difficult to change human behaviour when the built amenities do not support this behavior change [8].

These concerns have caused the architectural profession to expand their focus from spatial experiences, impressions, and “gut” feelings to include how physical environments can support public health [9]. However, this new approach is often based on traditional design strategies grounded in intuitive concepts and aesthetic considerations rather than scientific evidence that establishes clear relationships between the intent of the physical environment, and physical and behavioural outcomes [10]. It has been argued that the design profession must move towards a more “evidence-based” practice, with more explicit use of empirical evidence [11]. With influences from health science it is expected that designers will embrace additional sources of evidence to strengthen their design decisions in creating environmental conditions for specific user groups [12]. The interdisciplinary collaboration is mutually relevant for researchers whose objective is to increase the general activity level of the population [13].

Urban public spaces have become the focus of this discussion, particularly the inclusion of design elements that activate the end-users. Often the starting point for these efforts are classic sport facilities, such as football fields, skate spaces and street basketball. On paper, the strategies to transform and activate these settings tend to use trendy materials, such as rubber asphalt and flashy colours, according to the current fashion in landscape design [14,15]. These types of spaces primarily appeal to boys and young adult males, a generally active group, making it easy for designers to activate these settings [16]. These projects designed for a specific purpose are more likely to attract boys and young adult males who are already physically active, while nonspecialized spaces with an open use are more likely to attract those who are less physically active [17]. Recent research has focused on the activity level in parks, but the published outcomes do not point to specific design guidelines to attract different user groups [18,19]. In general, these studies have not explicitly stated any recommendations for architects and designers to follow to enhance physical activities among less active groups such as young girls, women, and older adults.

When a space is designed for a specific user group, it often has certain physical attributes that determine whether the intended user group will occupy the space, i.e., location, safety, lightning or seating [20]. Teenagers (14–20 years old) are generally more mobile, and do not want to spend time at certain urban public spaces if they are not engaged or entertained [21]. They will seek alternative spaces according to their needs. On the contrary, seniors are less mobile and for them soft values such as lightning, planting, accessibility and feeling safe are more important [22].

This study investigates the use patterns of two new urban public sites for sport and recreation, and how these sites reflect the needs of the intended user groups. The specific aims are to examine (1) how sport and recreation facilities in urban public spaces are used; (2) how the initial design objectives (the intent) align with the final outcomes (the reality); and (3) to investigate the specific design elements the architects incorporated to ensure the initial design objectives were met. It was hypothesised that the use of transparent evidence during the planning and development process would enhance the performance for the end users [10]. In order to rely on evidence, the information must be transparent, accessible and understandable, so others can make critical judgements about applicability to their setting. The context for this investigation is two newly built urban spaces for sport and recreation: Lüders parking roof and Guldbergs Plads in the suburbs of Copenhagen. These two spaces are similar in size and purpose of use, but the intended user groups differ.

## 2. Methods

### 2.1. Study Design

This study used a mixed methods approach. A combination of quantitative and qualitative methods were used to answer the research questions. The quantitative part consisted of systematic observations SOPARC (System for Observing Play and Recreation in Communities) [23], while the qualitative component consisted of semi-structured interviews with the responsible architects.

The study focused on two public urban spaces for physical activities; Lüders parking roof and Guldbergs Plads (hereafter referred to as spaces). The two spaces vary in terms of specific users, but are similar in size and intended purpose of use. Lüders parking roof was aimed at families with young children, while Guldbergs Plads was aimed at adults unfamiliar with physical activity. The definition of adults unfamiliar with physical activities was not defined in the objectives. The spaces are located approximately 4 km apart in two different suburbs of Copenhagen: Nordhavn and Nørrebro. The two suburbs have different populations in terms of age, ethnicity and socioeconomic factors. The spaces were chosen based on the intended use of each space.

### 2.2. Study Spaces

#### 2.2.1. Description of Lüders Parking Roof

Lüders parking roof is a new space for sport, play and recreation in Nordhavn. The space is placed on top of a parking structure 24 m above sea level, offering a great view across the harbour and city. The parking structure is placed in a dense newly built neighbourhood, and opened in August 2016. The inhabitants of this neighbourhood have high socio-economic status, primarily families and wealthy seniors. The 2500 m^2^ roof is open to the public and offers different activities such as CrossFit, Box jump, running stairs, Panna-football, trampolines and swings. It is possible to reach the roof from inside the structure, or on two staircases attached outside the parking structure. The space is divided into two areas, one for physical activity and another for play and recreation. A red metal bar separates different areas in waving shapes. The bar is raised approximately three meters above ground level. Ropes, swings and nets hang from the bar, offering different activities. The surface is covered with red rubber asphalt making the floor suitable for play and sport. Benches positioned along the edge offer seating overlooking the space. The development company, By & Havn (City & Harbour), commissioned the space, and it was financed in collaboration with The Danish Foundation for Sport and Cultural Facilities. The space was designed by an architect office called JAJA Architects. They were chosen through a competition, although the objectives for the use of the space were not described in detail before By & Havn had chosen the winning proposal.

#### 2.2.2. Description of Guldbergs Plads

Guldbergs Plads is a public space in Copenhagen in the district Nørrebro. The space can be dated back to 1915, and is surrounded by social housing. From 2013 to 2015 the run-down space went through a thorough renovation to offer new facilities for sport and recreation. Low socio-economic status, social tensions, and a high proportion of people with a non-western background characterize this neighbourhood. The objective for the renovation was to create at space for physical activity, especially for adults unfamiliar with physical activities.

The space is approximately 4000 m^2^ (including a 1000 m^2^ fenced dog space), and is surrounded by a low fence, but it is possible to enter the space through several gates. There is a connecting path going through the space, dividing the space into sub-areas. Three existing sub-areas, a fenced playground, a cage for ball games and a fenced area for dog walking, were left untouched. In the fourth sub-area, an existing lawn was turned into a landscape for play, recreation and physical activities. Throughout the space, ten small mounds with grass were added. Small clusters of blue vertical poles were spread across the space—200 in total. The poles offer different activities, such as swings, climbing poles, pullups or gymnastic rings. The surface is mainly grass, but rubber asphalt is also used on the biggest mound. Between the mounds and small trees, benches are placed randomly, offering secluded seating for relaxing. The space was commissioned by the Municipality of Copenhagen, and designed by the landscape architect company 1:1 Landscape in collaboration with the architect firm Keingart.

### 2.3. Office Interviews

To gain knowledge of the design process behind the two spaces, semi-structured interviews with the architects responsible for each space were conducted. The interviews were semi-structured because they followed a pre-prepared interview guide, but this could deviate if interesting themes appeared that were worth pursuing [24]. The interviews were conducted to see what they had done to achieve the initial objectives (the intent) and attract the expected user groups. The Lüders parking roof development company, By & Havn, who commissioned the project and established the objectives (the intent) were also interviewed.

An interview guide was developed containing a list of six questions. The guide focused primarily on three themes: (1) Design process; (2) Interdisciplinary collaboration; and (3) Owner evaluation of the space after completion. Examples of these questions include:

- Have you used interdisciplinary knowledge to enhance the design process?
- How did you reach the specific design, and end up with this solution?

Each interview took approximately twenty minutes and was conducted with the architects at their office. The “seven stages of an interview inquiry” by Kvale and Brinkmann [25] framed the organization of the interviews. These guidelines established the themes of the inquiry, the strategy for conducting the inquiry, conducting the interviews, analysing the data and verifying the results in terms of validity and generalization [26]. The interviews were recorded and transcribed in preparation for analysis.

### 2.4. Site Observations

Data were collected through site observations at the chosen urban spaces using the SOPARC framework. This is an objective observation method used to gather data about the users of a space. It is widely used as it is a reliable tool to collect physical activity data in parks or urban green spaces [16,27,28,29]. User groups and activity level had to be defined to collect data in a systematic way. The activity level was defined as three categories (sedentary, walk and vigorous), the purpose of the activity was defined in two categories (sport and play), and the user groups were defined based on observed gender, age: children (0–13), teenagers (14–20), adults (21–59) and seniors (60+), and whether the person was part of a group or an individual [30].

The observations were shared between two observers; the main author and a student assistant. A structured observation protocol was set up, and both observers took part in an observation training session prior to beginning. The observations were conducted over three periods; September 2016 (fall), January/February 2017 (winter), and April/May 2017 (spring).

**Figure 1:**
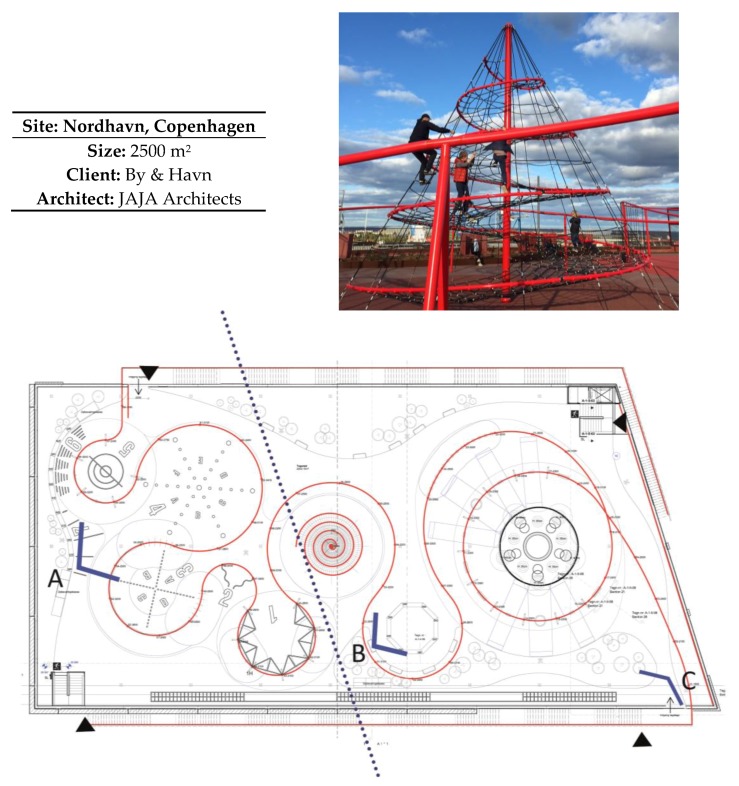
Overview of the Lüders parking roof. In order to make manageable observations, the space was divided into three sub-areas. Sub-areas A and B cover the roof, while sub-area C covers the staircase. The angle explains the field of view during the observations.

**Figure 2:**
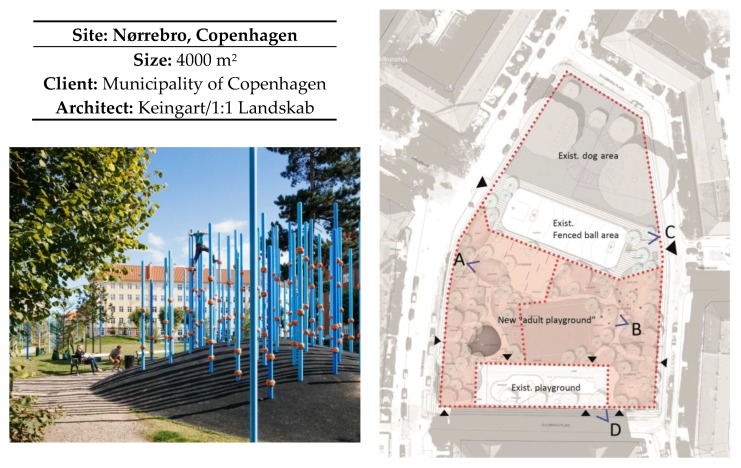
Overview of the Guldbergs Plads. In order to make manageable observations, the space was divided in to four sub-areas. Sub-areas A and B cover the new part of the park, while sub-area C covers the existing ball area and dog area. Sub-area D covers an existing playground. The angle explains the observer’s position and field of view during the observations.

There were 16 observations at each site, and observations were made four times per day during the following times: 7:30–8:30; 11:30–12:30; 15:30–16:30; 20:00–21:00. This took place three times during the week and once during the weekend. Lüders parking roof was divided into three sub-areas, and Guldbergs Plads was divided into four sub-areas shown in Figure 1 and Figure 2.

### 2.5. Data Analyses

The semi-structured interviews were typed and categorized in distinct categories to aid comparison. All SOPARC data were recorded in Microsoft Excel from handwritten observation sheets. The data were analysed using a descriptive methodology and graphics were used to visualize and illustrate associations and patterns in data. The mixed method gave the opportunity to explore questions with both a qualitative and quantitative approach.

## 3. Results

### 3.1. Lüders Parking Roof, Nordhavn

#### 3.1.1. Interview with JAJA Architects

JAJA Architects were invited to compete in a competition about the design of a new parking structure in Nordhavn by By & Havn. The office was competing against two other offices. Their interest in the project was based on the specifications of how the space should appeal to both residents, commuters, active or inactive users across all age groups.

In the competition phase, JAJA Architects introduced the concept designated the Red Thread. The intent of the Red Thread was to physically connect the space with a raised metal bar, three meters above ground level. In the competition phase, JAJA Architects divided the space into four subspaces; a ball cage, a climbing tower; a circular swing space and a circular water apparatus.

After JAJA Architects were selected as the competition winners, they focused on the specific design of the recreational and physical activity areas. During this phase, the office had 3–4 meetings with By & Havn and sport experts from DGI (Danish Association of Gymnastics and Sport Clubs) to determine the different attributes of the roof. JAJA Architects provided only a brief description of the specific attributes of the project, and together with DGI, they decided on the specific functionalities and design elements. The office presented their ideas and received input back from By & Havn and DGI. The overall layout stayed the same, but the functionality became more focused on CrossFit and weight training, since By & Havn had previous experiences from similar parking structures in Copenhagen. Therefore, By & Havn decided that running and training should be the primary functionality for the parking roof.

The Foundation for Sport and Cultural facilities (LOA) was later included in the design discussion of the parking roof, and they challenged the design of the ball cage functionality. They were questioning if the cage could have a multi-functional purpose. Eventually, the cage was removed from the layout. The process went back and forth, where the specialist gave input to the functionality and JAJA gave feedback represented by new design proposals. The architect stated: “DGI was mostly involved in the specific design of the elements, while LOA was more involved in the overall program of the space”. The involvement of DGI was most apparent in the beginning, but continued throughout the project. JAJA Architects agreed that they were not involved in the selection of the specific functions, because it was more or less prearranged. The design outcome was an evolving process and it *changed along the way.* JAJA Architects would have liked to evaluate the space, but had not laid out any specific plans. The architects said: “We often visit the space and make our own observations”.

The architect was not sure who established the objectives of the parking roof, and suggested that By & Havn may be able to provide further insight.

#### 3.1.2. Short Interview with the Project Manager at By & Havn (14 December 2016)

According to the project manager, there was a coherent agreement between the municipality of Copenhagen and DGI that ball cages are not especially useful. Therefore, the ball cage was abandoned from the project. Afterwards it was decided that the parking roof’s functionality should be running and CrossFit.

When asked about the origin of the initial objectives, the project manager said: “Maybe it’s not a question about the space being used the right way, but more a matter of the objectives being identified at the beginning”.

#### 3.1.3. Observations

**Figure 3:**
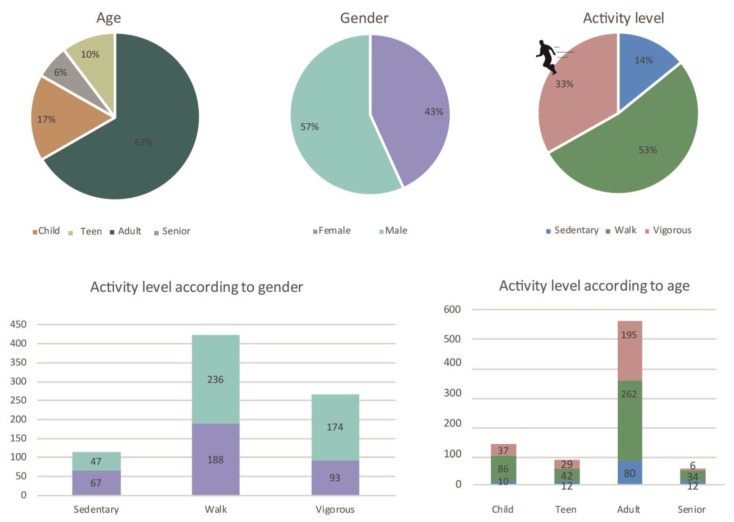
Observed age, gender and activity level on Lüders parking roof.

The space was divided into three sub-areas in order to make manageable observations. A total of 805 individuals (57% males/43% females) were observed at Lüders parking roof. This sample consisted of 17% children, 10% teens, 67% adults and 6% seniors (Figure 3).

According to activity level, 53% were moderately active, while 33% were vigorously active, and 14% were sedentary. There was a clear difference in activity level between gender; 174 males (38% of all males) were vigorously active, while only 93 women (27% of all women) were active at a vigorous intensity. Of the 267 individuals that were vigorously active, 218 (82%) used the facilities the intended way, either for running or working out on or around the red bar.

Although the overall proportion of males and females was similar, there were prevailing differences when investigating sub-areas of the space. Sub-area A is designed for sport and most users in this area were males (167 males/98 females). Among those who were vigorously active, the difference was more pronounced: (89 males/44 females).

**Figure 4:**
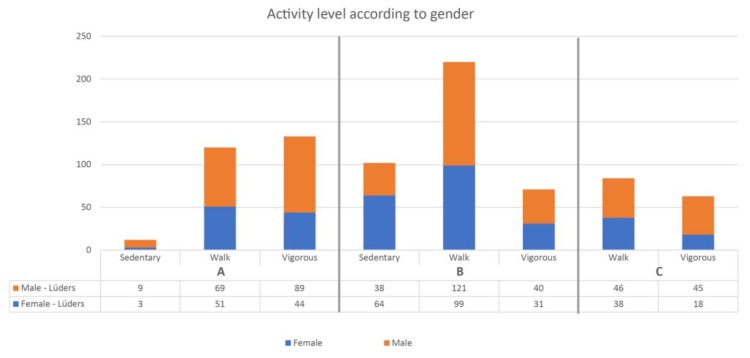
Observed gender distribution across the sub-areas of the space. It can be seen that there is the most vigorous activity in sub-area A.

In area B, which is designed for play and recreation, the numbers were more similar with 199 males and 194 females. In area C there was mostly sedentary females (38 males/64 females) (Figure 4).

### 3.2. Guldbergs Plads, Nørrebro

#### 3.2.1. Interview with Keingart Architects (26 August 2016)

The project initially attracted attention from the architects because the objective was to develop a strategy to enable the physically inactive users to become physically active users. As adults who were unfamiliar with physical activity were the target group, the architects considered traditional fitness equipment unsuitable as it might intimidate this group from visiting the space. Therefore, the focus was to create an appealing space that could generate physical activity in other ways.

Local residents’ input was prioritized, because the architects wanted to avoid a space that resembled an outdoor gym, as placed elsewhere around Copenhagen. To reveal their ideas and ambitions, Keingart invited the potential user group on a study tour to visit similar projects in Copenhagen. The users independently evaluated each space during visits using a checklist. This data provided the architects with a snapshot of which facilities the user group appreciated the most. The users primarily wanted equipment that was fun, such as swings, and it was important that each element was easily accessible. This lead to the architects’ design principle: It should be possible to access the space with two shopping bags in your hands.

The overall design of the space was grounded on aesthetic considerations. The specific elements were designed in collaboration with a physiotherapist so the size and position of the equipment was suitable for overweight and older adults. Keingart participated in the project until the construction phase. In hindsight the architects stated that some of the initial design ideas were lost after they left the project. An example could be the size of swing seats designed for adults, but built for children.

The architects had the intention to evaluate the site but did not succeed due to lack of time and money. However, the architect emphasised the importance of an evaluation: I think it could be interesting, and it would be great, if it was possible to convince the client that they should allocate money to do an evaluation afterwards.

#### 3.2.2. Observations

**Figure 5:**
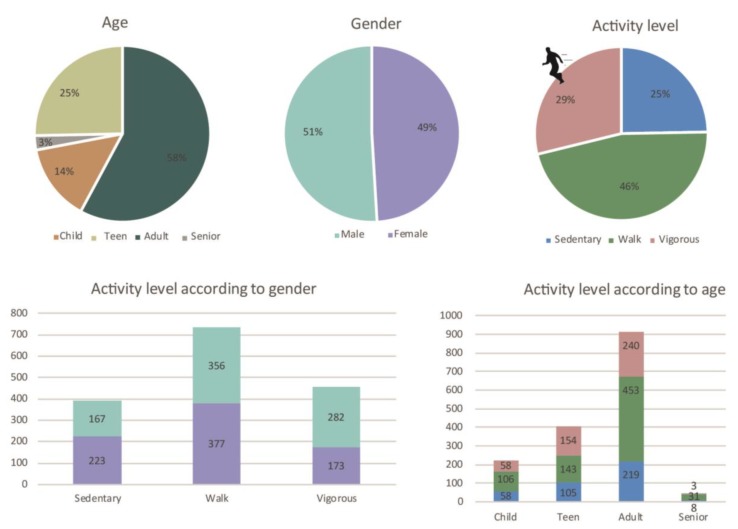
Observed age, gender and activity level on Guldbergs Plads.

The space was divided into four sub-areas in order to make manageable observations. A total of 1658 individuals (51% male/49% females) were observed on Guldbergs Plads. This sample consisted of 14% children, 25% teens, 58% adults and only 3% seniors (Figure 5).

According to activity level, 46% were moderately physically active, while 29% were vigorously active and 25% were sedentary. There was a clear difference in activity level between gender; 282 males (35% of all males) were vigorously active, while only 173 women (22% of all females) were active at a vigorous intensity.

**Figure 6:**
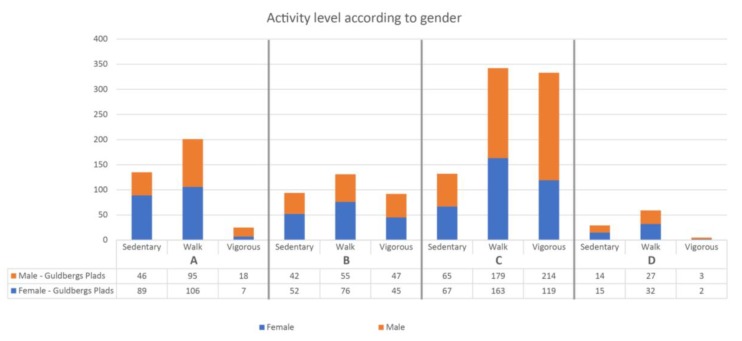
Observed gender distribution across the sub-areas of the space. It can be seen that there is the most vigorous activity in sub-area C.

In sub-area A. there were twice as many sedentary females compared to males, and more than twice as many males who were vigorously active. The numbers of males and females were almost the same in sub-area B, which was designed for physical training. In contrast, sub-area C (where the ball cage is placed) had almost twice as many vigorous males (214 males/119 females) (Figure 6).

A total of 255 people used Guldbergs Plads for vigorous play and sport (not including the people passing the space by bicycle). Of these, 140 were inside the ball cage, and 115 were vigorously active in sub areas A and B. A total of 35 people used the bars for play and working out. Only seven adults used the bars, and no seniors were observed using them. Most of the vigorously active individuals used the ball cage for ball games.

### 3.3. Comparison of Lüders Parking Roof and Guldbergs Plads

**Figure 7:**
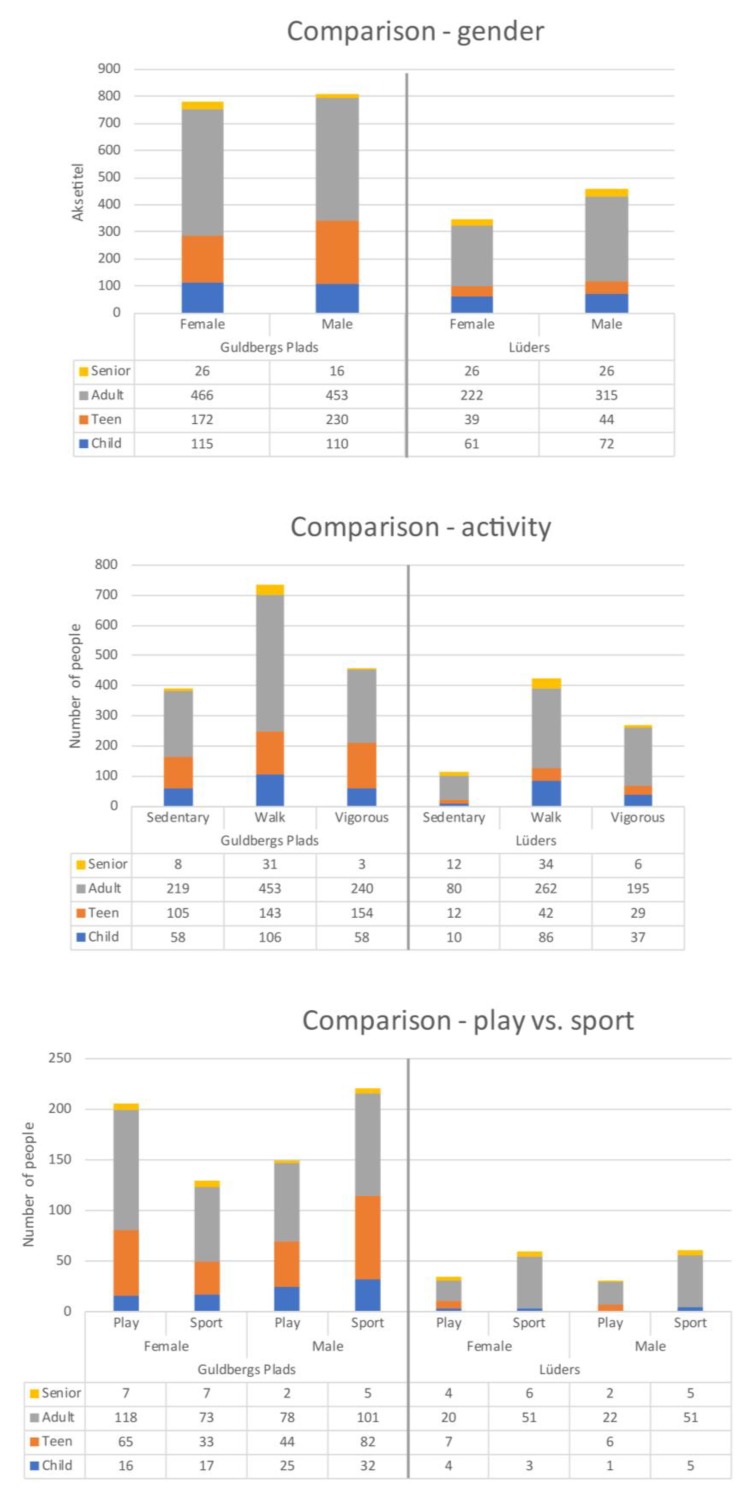
A comparison between Lüders parking roof and Guldbergs Plads regarding gender, activity and play vs. sport.

In this study, a total of 2463 users were observed at the two spaces: 805 users at Lüders parking roof and 1658 users at Guldbergs Plads. The variation in age groups and gender was diverse for both Lüders Parking Roof and Guldbergs Plads, but similar between sites. However, Guldsbergs Plads had a larger proportion of adolescents compared to Lüders parking roof. Most teenagers were males (230 males/172 females) due to the ball cage at Guldbergs Plads, where the males play football and other ball games. No females were observed playing ball games in the ball cage. On Lüders Parking Roof there was a clear majority of male adults (315) compared to females (222). This was due to the parking roof offering very specific physical training in sub area A (See Figure 7).

When looking at activity level, there were only few sedentary people on the parking roof. Only 114 sedentary people out of 805 observed people. This can be explained by the fact that the roof is designed for physical training, and therefore appeals less to people with limited training experience. It is noteworthy that there were many adolescents at Guldbergs Plads while there were only a few at Lüders Parking Roof. This may be explained by the initial objectives for Lüders Parking Roof, which was aimed at families with small children (Figure 7).

## 4. Discussion

The overall aim of this study was to investigate how two urban public spaces for sport and recreation were aligned with the initial objectives (the intent) and to what extent the spaces reflected the needs of the user groups (reality). The SOPARC observation data showed that both sites have a diverse user profile. The intended user groups of Lüders parking roof were families with young children, while Guldbergs Plads was aimed at adults unfamiliar with physical activity. The user groups were not defined more specifically than this, so it was difficult to point out the adults unfamiliar with physical activity compared to adults used to physical activity. At first glance, the total numbers suggest both design processes can be considered successful. More in-depth analyses, however, gave a varied picture showing a discrepancy in initial objectives and actual use. The parking roof data revealed that male adults and boys used the parking roof more than women, girls and adolescents. Almost no children were observed in sub-area A, most likely because children could not reach the three-meter raised red bar. This is a clear example of a facility not aligned with the initial objectives; to design facilities that attract families with small children.

At Guldbergs Plads, most users were active male adults and male adolescents, but only a few were observed using the blue poles for physical activities, and no adults or seniors were observed using the poles. This is in contradiction to the initial objective that targeted adults unfamiliar to physical activities. The architect explained that the target group were “scared” of failing and making fools of themselves. To avoid this the architect had described wide swing seats to make them suitable for overweight users. Through the construction phase, this request vanished, and the swings were assembled with small, narrow seats meaning they went unused by the intended users. This is another example of obstructions in delivering the initial objectives to end users. The architect expressed the loss in translation with his own understanding of the requirement: Some functions were lost along the way, since the history is forgotten through the development process.

A key premise is that knowledge of how site attributes impact the use of urban public spaces for physical activity is essential to develop and make the appropriate design decisions. According to the interviews, the architects tried to make the public spaces available for a wide user group, and a way to secure this is by using interdisciplinary collaboration. Architect and theorist John Habraken pointed out that there is a gap between the initial objectives and the factual use of facilities [31]. He also questioned whether architects base their solutions on interdisciplinary knowledge as a means to strengthen the basis for design decisions. Today it is too easy for architects to decide what kind of interdisciplinary expert knowledge they incorporate into new projects based on their own “expert” intuition [31]. When looking at the ecological model of four domains of physical activities, one can conclude that good design cannot change the behaviour of people by itself, but other factors must also be taken into consideration. Therefore, there is an urgent need for a rigorous method that can both systemize and priorities the kind of interdisciplinary expert knowledge necessary to enhance the use of new facilities.

In a review of facilities built for exercise and movement, Wikke and Skoubøll provide examples of how successful research has been integrated into designing indoor facilities for yoga, pilates and fitness; however, no such examples exist for outdoor spaces [32,33]. To our knowledge, a large number of urban, public sites are primarily designed using professional experiences and casual subjective knowledge (expert intuition). We hypothesized that this design approach poses a high risk of designing sites with mere aesthetics and low user-friendliness.

Designing to activate certain end-users is a challenging task, especially groups with limited activity experiences in a public setting and low self-esteem. For the same reasons some groups use urban spaces more frequently than other groups [34]. Traditional methods to inform the design process are widely used, mostly due to a lack of transparent knowledge in the design profession making it difficult for architects to learn from similar projects and bring this knowledge into their own design process. In addition, there is a lack of time and resources for conducting interdisciplinary collaboration. According to the ecological model of four domains of physical activities, good design determines physical activity behaviour but other factors relating to policy, environmental context, demographics and individual values, beliefs, and traditions should be included as parameters in the design assessment. This broad perspective, however, puts demands on interdisciplinary knowledge that exceed what a single specialist can comprehend and deliver. The interviews revealed that the architects had not used any systematic interdisciplinary collaboration. In both projects, the architects had invited sport specialists to inform the design process, but other aspects e.g., accessibility and safety, were not taken into consideration. Studies have shown that less programmed public spaces with open-ended facilities are more likely to attract users who are less physically active [17], so both sites would have gained by drawing on disciplines other than sport science, such as environmental psychology.

It could be claimed that the initial objectives were not aligned with the local residents’ needs. The Foundation for Sport and Cultural facilities (LOA), who partly financed the parking roof, has a funding policy to support multi-functional facilities based on evidence. The funding strategy also supports new innovative projects that create extraordinary spaces and extraordinary design with an overall purpose to inspire and develop traditional sport architecture and urban planning. This leads to projects appearing as “ahead of their time”, but as a consequence can be out of sync with both owners’ and end-users’ needs. The initial objectives for Guldbergs Plads were apparently not based on any previous research, but a political decision to solve public health issues amongst the inhabitants of Nørrebro.

In the research paper Active use of urban park facilities—Expectations versus reality the authors stress the demand for political involvement to change the planning process of new spaces for physical activity. Lindberg and Schipperijn highlight that the planning process should embed the newest available evidence-based knowledge in future planning of facilities in urban green spaces [27]. The research paper Translation active living research into policy and practice suggests ten strategies that may help translating active living research into policy and practice. One of the strategies includes interdisciplinary research teams [13]. This finding supports the hypothesis of this study that the use of transparent evidence in the planning and development process will enhance the performance for the end users. The analyses in our study underpin the need to develop rigorous methodological approach to optimize the conditions for successful construction of future spaces for sport and recreation. The method should draw on knowledge between interdisciplinary fields. There are only a few examples of evidence-based design used to reconcile specific architecture with certain forms of movement [35], and some research has been carried out on playgrounds specifically [36]. An explanation for limited research is that the topic is placed between different disciplines, such as architecture, sport, design, physical culture and environmental psychology. Therefore, a broad interdisciplinary scientific approach is required.

Architects do not traditionally conduct systematic evaluations of their finished projects. Typically, they visit the sites and observe the use of the space. If the space is occupied it is assessed as a success regardless of the specific use of the space. The first observations of Lüders parking roof were conducted after the initial opening of the space. We were aware that the publicity of the opening could lead to an increased number of visitors. A comparison of the use during various seasons also revealed that there was a big drop off for Lüders parking roof compared to Guldbergs Plads. From fall to spring there was a drop of 12.4% for Guldbergs Plads and a dramatic drop of 80.5% for Lüders parking roof. JAJA architect indicated in the interview that they often visited their own projects to make self-evaluations. During the first SOPARC observation in the fall of 2016, shortly after the opening, a lot of people visited the parking roof, but according to the observation notes, these people were just observing the new space out of curiosity. The news interest will have a positive effect on the architects’ self-evaluation, but not necessarily over a longer period.

This study used two methods to analyse the two spaces: SOPARC and semi-structured interviews of the involved architects. SOPARC does not examine user satisfaction with the two spaces, but only answers two questions: who and when. Therefore, it could have been interesting to conduct an additional interview or questionnaire that investigated how and why the users were active in each space. The semi-structure interviews provide reliable, comparable qualitative data, but the risk is that the open-ended questions can be difficult to analyze. Furthermore, it is difficult to guarantee the reliability of the participant’s stories, as the research process focused on events and outcomes from the past.

Furthermore, this research paper did not focus on cultural and social differences, such as age, ethnicity and socioeconomic factors at the two spaces, but these may affect the generalisability of these findings. In future studies it could be interesting to include these parameters.

## 5. Conclusions

Overall, the two spaces were successful in attracting users, but the users did not align with the initial target groups. The architects responsible for designing each space did not integrate the right interdisciplinary collaboration at the right time during the planning process and did not have a rigid method to systematically integrate this into the design process. According to the ecological model, architects must incorporate many different factors to fulfil the future needs for activating architecture. This means that architects must change their focus from aesthetics to a more evidence-based approach.

The intent of this research paper was not to point out the reasons why the architects did not succeed, but to call for a more systematic critical method as a means to bridge the gap between the initial objectives (the intent) and the everyday use of specific space (the reality). Such a method will introduce a more systematic design process where interdisciplinary knowledge is integrated into the design process. This may enhance activity levels in urban public spaces, and improve the health and wellbeing of the local community.
